# Profiling the Longitudinal Development of Babbling in Infants with Cerebral Palsy: Validation of the Infant Monitor of Vocal Production (IMP) Using the Stark Assessment of Early Vocal Development-Revised (SAEVD-R)

**DOI:** 10.3390/diagnostics13233517

**Published:** 2023-11-23

**Authors:** Roslyn Ward, Neville Hennessey, Elizabeth Barty, Robyn Cantle Moore, Catherine Elliott, Jane Valentine

**Affiliations:** 1 Department of Paediatric Rehabilitation, Perth Children’s Hospital, Perth 6009, Australiacatherine.elliott@telethonkids.org.au (C.E.); jane.valentine@health.wa.gov.au (J.V.); 2 School of Allied Health, Curtin University, Perth 6102, Australia; n.hennessey@curtin.edu.au; 3 NextSense Institute, Sydney 2151, Australia; robyn.moore@nextsense.org.au; 4 Faculty of Arts, School of Education, Macquarie University, Sydney 2109, Australia; 5 Telethon Kids Institute, Perth 6009, Australia

**Keywords:** cerebral palsy, babbling development, Infant Monitor of vocal Production, clinical utility

## Abstract

Aim: We compared early vocal development in children “at risk” for cerebral palsy (CP) with typically developing (TD) infants aged 6 to 15 months using the SAEVD-R, investigating potential pre-linguistic markers of communication impairment. Additionally, we sought to examine the agreement between the SAEVD-R and IMP, which uses parent report, in identifying departure from typical vocal development in at-risk infants. Method: Utilising a longitudinal cohort study, >10,000 vocalisations of 33 infants (15 at risk for CP and 18 TD) were assessed at 6, 9, 12, and 15 months using the SAEVD-R. Generalised linear mixed models (GLMMs) compared groups, and Spearman correlations explored IMP ceiling scores and SAEVD-R measures. Results: At 6 months, both TD and CP groups reached SAEVD-R vocalisation level 3 (expansion). By 9 months, 51% of TD infants progressed to advanced babbling (levels 4 and 5), while 80% of at-risk infants remained at level 3. At 12 and 15 months, over 90% of TD children advanced, compared to 67% at 12 months and 53% at 15 months for at-risk infants, who stayed at the pre-canonical stage. Strong correlations were found between IMP scores and vocalisation levels at 9 and 12 months. Remaining at the pre-canonical stage at 12 months correlated with delayed vocal development as per IMP scores. Interpretation: TD infants achieved higher SAEVD-R levels than at-risk infants. At 12 months, IMP scores effectively identified infants with speech-like vocalisation difficulties, demonstrating its clinical utility in identifying atypical vocal development in infants at risk for CP.

## 1. Introduction

Reliable and valid assessment tools for early identification of cerebral palsy (CP) have enabled practitioners to identify CP in infants younger than 6 months of age with high specificity and sensitivity [1], expediating judicious referral to CP-specific interventions focused on optimising developmental outcomes [2,3]. In contrast, there is a notable absence of tools with established psychometric properties for the early identification of communication impairment. Current prediction models of communication impairment are reliant on assessments conducted at or after two years of age [4,5,6]. Critical periods of development within the first 24 months are, therefore, being missed, ultimately delaying access to targeted early intervention. 

The first 12 months of life are associated with a significant period of growth in speech and language development [7]. During this period, volitional speech-like vocalisations, known as protophones, emerge following a developmental sequence of progression from basic vocalisations to increasing complexity [8,9,10]. These volitional vocalisations are considered developmental precursors to children’s first words, showing “continuity” between the speech sounds expressed in these vocalisations and spoken words [11]. 

Research suggests early departure from typical development in the use of early protophones may be a valid predictor of expressive language skills not only in neurotypically developing children but also in children with neurodevelopmental conditions [12,13,14]. Marked differences in the vocalisations of infants with neurodevelopmental disorders have been reported, including autism spectrum disorder [15], Down syndrome [16], fragile X [17], and CP [18,19,20,21]. Of interest to this paper, children with CP have been shown to differ in their ability to use different sounds together and babble fluently and rhythmically [19,21]. These findings suggest identification of communication impairment secondary to CP is possible before 2 years of age and indicate the need for clinically feasible and valid assessment tools.

### 1.1. Assessing Infant Vocalisations

The human coding of infant vocalisations is considered fundamental in characterising developmental differences and deviation from typical pre-linguistic vocal development [9,22]. It entails the determination of utterance and vocalisation boundaries, as well as the classification of protophone types and characteristics. This is typically undertaken using coding schemes based on staged models of development [9,23], such as the Stark Assessment of Early Vocal Development-Revised (SAEVD-R, [8]). 

The SAEVD-R is grounded in a motoric perspective with a focus on “articulatory complexity” [9] and provides operational definitions for 23 types of vocalisations, classified into five levels: at the *reflexive* level (0–4 months), vocalisations include vegetative sounds, such as fussing and crying, which are not under voluntary control, as well as the quasi-resonant nuclei protophones. The *control of phonation* level (1–4 months) represents sounds under voluntary control, including vowel-like and consonant-like sounds (such as raspberries). The level of *expansion* (3–8 months) marks the transition to mature vowels that are transcribable. This includes marginal babbling, characterised by a sequence of consonant-like and vowel-like syllables, with a transition time that is slower than evidenced in canonical babble. The *basic canonical* level (5–10 months) involves the production of syllables containing a consonant and vowel (CV), with rapid transitions. The *advanced forms* level (9–18 months) marks increased complexity in motor control including the production of CVC syllables, consonant clusters, and the emergence of first words [8,24].

The human coding of infant vocalisations is, however, time intensive, expensive, and requires specialist training [9]. For this reason, human coding is typically limited to the research environment [9,25,26], thus limiting the clinical assessment of infant vocalisations and potentially delaying the early identification of communication impairment.

### 1.2. Assessment of Infant Vocalisations within the Clinical Setting

In contrast to human coding, the literature indicates parent-reported measures provide an ecologically valid and cost-effective means to identify early departure from typical development [27,28]. To date, most parent-report measures have focused on the identification of canonical babble [22,29], the current accepted benchmark as a robust predictor of communication impairment [14]. Canonical babble not only precedes the development of spoken words [22,30], it is also reported that mothers are more responsive to infants who babble CV syllables than when vowel syllables alone are produced [31,32,33].

More recently, however, attention has also been directed at researching the potential predictive value of earlier-developing protophones [9,32,34] for even earlier identification of communication impairment. Nathani and Oller [9] suggest the pre-canonical stages may “yield predictive power comparable to that which has been found with the onset of canonical babble” (p. 322), which has relevance to early identification of communication impairment in the CP population. To this end, Ward et al. [21] reported on the assessment of infant vocalisations using the Infant Monitor of vocal Production (IMP, [28,35,36]) as a potentially predictive tool for use within the clinical environment. 

The IMP was found to be sensitive to departure from typical development in children identified as at risk of communication impairment secondary to CP [21]. Specifically, the rate of development of more complex and speech-like vocal behaviours in the infants at risk of CP was slower compared to typically developing infants, with group differences emerging at 9 months and 12 months of age, when pre-canonical and canonical babble typically emerge. This suggests the potential for the IMP to be used to identify prelinguistic markers of speech–motor impairment, the most common form of verbal communication disorder in children with CP [37]. This evidence, however, was limited to the analysis of individual items from the IMP. It was shown that those items targeting the emergence of speech-like vocal behaviours, including canonical babbling, discriminated between the at-risk infants and typically developing infants, but not items reflecting vocal behaviours associated with social communication. There was, however, no independent corroboration of the findings through direct observation of the children’s vocal behaviour.

In this present study, we extend the findings of Ward et al. [21] through human coding of infant vocalisations captured via naturalistic all-day audio sampling [22] using infants from the Ward et al. [21] study as participants. These data were captured at the same time as the parent was interviewed to administer the IMP [21,35,36].

The purpose of this longitudinal study was twofold. First, we aimed to characterise and compare the development of early vocal behaviours across the 6 to 15 months age range in children identified “at risk” of CP with age-matched typically developing (TD) infants using the SAEVD-R [8] to investigate possible pre-linguistic markers of communication impairment. Secondly, we aimed to determine whether infant vocalisations coded using the SAEVD-R correlate with vocal development at 12 months of age based on performance on the IMP, as reported in Ward et al. [21]. This has implications for the criterion-related validity of the IMP in identifying impaired speech development in infants at risk of CP [35]. 

The following research questions were addressed:

1. Do the number, percentage, and level of vocalisations of children at risk of CP differ from TD children at 6, 9, 12, and 15 months of age, as measured on the SAEVD-R?
2. Is there agreement between the IMP and the SAEVD-R in identifying departure from typical vocal development in children at risk of communication impairment secondary to CP?

## 2. Method

### 2.1. Study Design

A case–control prospective longitudinal cohort study design was utilised. Approval for this study was obtained from the Child and Adolescent Health Services Ethics Committee of Western Australia, Perth, Australia (study number 2015221).

### 2.2. Participants

The participants of this study represent a subset of the 36 infants reported by Ward et al. [21]. We report on the vocalisations of 15 infants identified as “at risk” of CP at the time of enrolment into the study and 18 TD children, recruited between March 2016 and April 2019. Three at-risk participants reported by Ward et al. [21] were unable to be included in this study because audio recordings were not available. Table 1 provides a summary of the participant characteristics.

### 2.3. Setting 

Participants were recruited as part of a larger longitudinal study evaluating the development of communication in children aged 6 months to 2 years through the Early Intervention service of Perth Children’s Hospital (PCH). This is the state-wide tertiary rehabilitation service in Western Australia and designed to provide interim specialist multi-disciplinary early intervention services as described by Davidson et al. [38]. Data were collected within the home environment.

### 2.4. Materials

#### 2.4.1. LENA DLP 

Data were obtained through all-day recordings in the infant’s home, using the Language Environment Analysis (LENA^®^, Louisville, CO, USA) digital language processor (DLP). The LENA technology audio records and automatically calculates adult word count, conversational turn-taking, and speech-related vocalisations of infants in the home environment for up to 16 h, at a sampling rate of 16 kH [39]. The device is small in size and light and is worn in the pocket of a specially designed t-shirt, maintaining a consistent distance of the microphone from the infant’s mouth across recording occasions. Only audio recordings were used in the present study.

#### 2.4.2. Infant Monitor of Vocal Production (IMP)

The IMP is a normed, criterion-referenced instrument for infants from birth to 12 months of age that uses a semi-structured interview format to discuss with the parent the child’s level of achievement in relation to 16 vocal behaviours, with each behaviour rated using a 5-point Likert scale (Never = 0, Rarely = 1, Sometimes = 2, Often = 3, Always = 4). The target vocal behaviours (e.g., blows raspberries, able to combine different sounds, babbles fluently and rhythmically) are ordered in terms of increasing complexity similar to the SAEVD-R levels, from reflexive sounds and early vowel production to more speech-like canonical babbling and complex syllable sequences. 

The IMP has established psychometric properties [35] showing strong inter-rater reliability and correlation for agreement (0.94). The ceiling score, reflecting the highest level of vocal behaviour achieved by the infant, is sensitive to age-related changes in vocal behaviour in TD infants up to 12 months of age, as well as variations in progress towards speech in infants with hearing impairment [35,36] and motor impairment [21].

### 2.5. Procedure

Parents were provided with the DLP on the same day as the home visit that was undertaken to conduct the interview-based assessment of infant vocal behaviours, using the IMP [21]. Parents were requested to activate the DLP on a designated recording day, within 7 days of each home visit at 6, 9, 12, and 15 months of age. The DLP was inserted into the pocket of the LENA t-shirt as soon as the infant awoke on the morning of the designated day, with continuous wearing of the device throughout the day until evening bedtime. Parents were asked to maintain a log of the day’s activities and provide a 1-h period from within which time a 15 min sample (3 × 5 min) of the infant’s vocalisations could be extracted for analysis. Criteria for selection of the 1 h segment included: consenting participants present/recorded only, no personal conversations recorded (e.g., phone calls), limited background noise, and evidence of infant vocalisations. 

#### Coding and Measurement

The DLP data were transferred to the LENA Pro V3.50 144 r12080 software for extraction of the audio files as WAV files. The principal researcher extracted the WAV files and de-identified and randomised the speech samples. A second researcher undertook the coding (EB). The coder is a speech and language pathologist with a master’s degree in linguistics and more than 30 years’ paediatric clinical experience. The coder was blinded to the participant’s age and diagnosis, with audio samples randomised. 

The coder listened to the audio samples and visually inspected the waveforms and broadband spectrograms in Praat [40], then determined the onset and offset boundaries for each utterance and vocalisation. An utterance was defined as a vocalisation or group of vocalisations within one “breath group”, separated by audible breaths or pauses of 1.5 s or longer. Onsets and offsets of the infant vocalisations were located and marked in a Praat textgrid, with speech-like vocalisations coded using broad phonetic transcription. Repeat-listen coding was adopted [22].

Each vocalisation type within an utterance was coded using the operational definitions of SAEVD-R [8]. Each utterance was then assigned to one of the developmental levels (reflexive, controlled phonation, expansion, basic canonical syllables, and advanced forms). As per the protocol of Nathani et al. [8], “the level of the utterance was equal to the highest level associated with an individual vocalisation type within the utterance” (p. 8).

### 2.6. Data Analyses

The vocalisations or protophones in a total of 10,555 utterances were transcribed. The number and percentage of utterances, coded according to the protophone level of the SAEVD-R, were analysed, as well as the number and percentage of individual vocalisations (protophones). We analysed individual vocal behaviours, the protophones, and not just coded utterances to capture more fully between-group differences within utterances in the measurements. 

Generalised linear mixed modelling implemented with GENLINMIXED in SPSS Statistics (Version 28) was used to compare the at-risk and TD infants in the number and percentage of coded utterances and vocalisations separately for each SAEVD-R protophone level (i.e., reflexive, control of phonation, expansion, basic canonical syllables, advanced forms). Group (at-risk vs. TD), time (6, 9, 12, 15 months), and the interaction between group and time were tested as fixed effects and participants were included as random effects in the model. GLMM is suitable to analyse repeated measures designs and can handle missing cases and non-normal data distributions. An alpha level of 0.05 was adopted, and planned comparisons between groups at each time point were undertaken using sequential Bonferroni adjusted contrasts. Follow-up comparisons of main effects (e.g., of time) and interaction effects were undertaken using least significant difference contrasts. Partial eta squared effect size statistics, obtained from general linear model analysis in SPSS, are reported for the fixed effects analyses. 

## 3. Results

### 3.1. Do the Number, Percentage, and Level of Vocalisations of Children at Risk of CP Differ to TD Children at 6, 9, and 12 Months of Age, as Measured on the SAEVD-R?

#### 3.1.1. Total Number of Utterances and Vocalisations

See Table 2 for the mean total number of utterances at each time point for at-risk and TD infants. GLMM analysis showed an effect of time, *F*(3, 119) = 6.061, *p* < 0.001, η_p_ = 0.16, but no significant group effect, *F* < 1, or interaction between group and time, *F*(3, 119) = 1.832, *p* = 0.145, η_p_ = 0.04. Contrast analysis showed the marginal mean total number of utterances at 9 months (69.6 ± 3.2) was significantly lower than the 6-month (86.2 ± 3.6), 12-month (82.6 ± 4.4), and 15-month (85.7 ± 4.6) marginal means, *p* < 0.05. 

For the total number of vocalisations (Table 3), GLMM analysis showed at-risk infants produced significantly fewer vocalisations than TD infants, *F*(1, 119) = 6.189, *p* = 0.014, η_p_ = 0.18. While there was no effect of time, *F* < 1, there was a significant interaction between group and time, *F*(3, 119) = 3.528, *p* = 0.017, η_p_ = 0.08. The mean total number of vocalisations for at-risk infants was significantly lower than TD infants at the 6-month *t*(119) = 3.194, *p* = 0.002, and 15-month time points, *t*(119) = 3.098, *p* = 0.002, but not at 9-month, *t*(119) = 0.095, *p* = 0.925, or 12-month time points, *t*(119) = 0.851, *p* = 0.396.

#### 3.1.2. Number of Protophones

**Figure 1:**
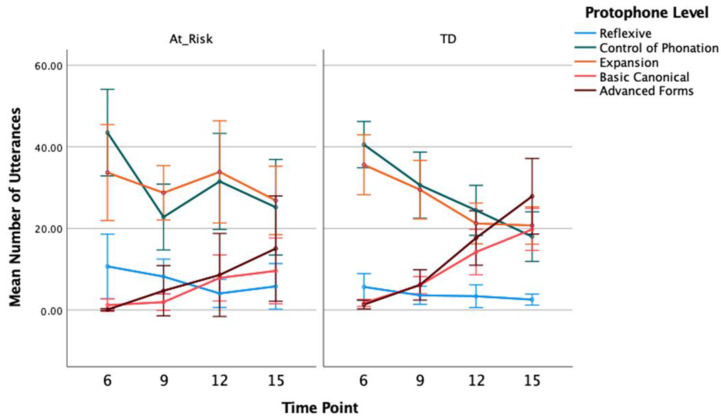
Mean Number (With 95% CI) of Infant Utterances Coded as Reflexive, Phonation Control, Expansion, Basic and Advanced Canonical for At-Risk and Typically Developing Children at 6, 9, 12, and 15 Months of Age.

**Figure 2:**
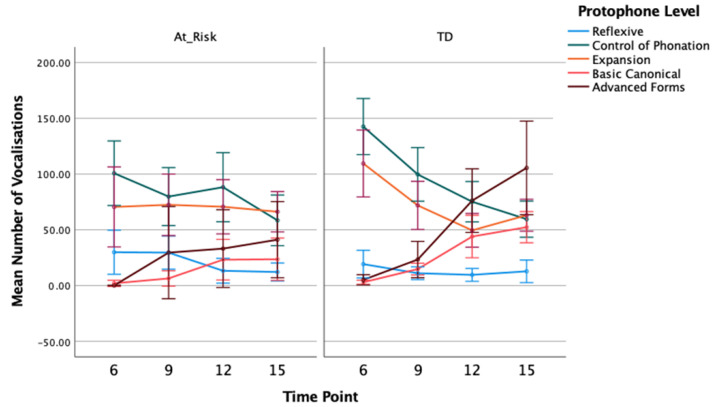
Mean Number (With 95% CI) of Infant Vocalisations Coded as Reflexive, Phonation Control, Expansion, Basic and Advanced Canonical for At-Risk and Typically Developing Children at 6, 9, 12, and 15 Months of Age.

The mean number of utterances and vocalisations coded at each protophone level, split by group and time, are included in Table 2. Visual displays of the timelines for each group with 95% confidence intervals are in Figure 1 and Figure 2. As a general pattern, the most common protophones at the youngest age of 6 months are the pre-canonical control of phonation and expansion type level sounds, followed by reflexive sounds. There were comparably few basic canonical sounds produced and advanced sounds are largely absent. For TD, in particular, there is a reduction in the pre-canonical sounds across the time points up to 15 months of age and a corresponding increase in basic canonical and advanced forms. These changes are evident in the percentage data reported below, as well. For the at-risk infants, there are some exceptions to this overall pattern, however, with differences supported by the results of the GLMM analyses as follows. 

For the number of reflexive level utterances, GLMM analysis showed a main effect of time, *F*(3, 119) = 4.490, *p* = 0.005, η_p_ = 0.08, with a trend for the number of reflexive utterances to decrease at later time points (12 and 15 months) compared to earlier time points (6 and 9 months), but no overall group effect, *F*(1, 119) = 3.039, *p* = 0.084, η_p_ = 0.09, or group by time interaction, *F*(3, 119) = 1.409, *p* = 0.243, η_p_ = 0.02 (Figure 1). 

Similar results were found for the number of reflexive vocalisations; the mean number of reflexive vocalisations across both groups reduced over time, *F*(3, 119) = 3.572, *p* = 0.016, η_p_ = 0.08, but there was no group, *F*(1, 119) = 3.736, *p* = 0.056, η_p_ = 0.08, or group by time interaction, *F*(3, 119) = 2.162, *p* = 0.096, η_p_ = 0.05 (Figure 2). The near-significant group effect appears to be due to a larger overall number of reflexive type vocalisations for at-risk infants compared to TD infants. Contrast analysis with adjusted p values showed the group difference at 9 months of age was statistically significant, *t*(119) = 2.563, *p* = 0.012. Descriptively, as shown in Figure 2, there was a reduction in the number of reflexive sounds produced at 9 months compared to 6 months for TD infants, whereas at-risk infants did not show a reduction in reflexive sounds until after 9 months. This delay in the decline of reflexive sounds for at-risk infants may have contributed to the group difference at 9 months, in particular. 

Analysis of utterances coded as control of phonation showed a significant decline over time, *F*(3, 119) = 13.528, *p* < 0.001, η_p_ = 0.33, as well as no group main effect, *F* < 1, and no significant interaction between group and time, *F*(3, 119) = 2.605, *p* = 0.055, η_p_ = 0.07. This pattern was also seen in the number of control of phonation type vocalisations: time as a main effect was significant, *F*(3, 119) = 15.603, *p* < 0.001, η_p_ = 0.31, but not group, *F*(1, 119) = 1.958, *p* = 0.164, η_p_ = 0.05, or the interaction between group and time, *F*(3, 119) = 2.365, *p* = 0.075, η_p_ = 0.08. 

For the number of expansion type utterances, there were no significant main effects of group, *F*(1, 119) = 2.551, *p* = 0.113, η_p_ = 0.07, and time, *F*(3, 119) = 2.438, *p* = 0.068, η_p_ = 0.08, and no group by time interaction, *F*(3, 119) = 1.185, *p* = 0.318, η_p_ = 0.06. There was also no group, *F* < 1, and no time effect, *F*(3, 119) = 2.552, *p* = 0.059, η_p_ = 0.09, for the number of expansion type vocalisations, but there was a significant group by time interaction, *F*(3, 119) = 3.450, *p* = 0.019, η_p_ = 0.12. The mean number of expansions reduced after 6 months for the TD group, with contrasts between the 6-month and all later time points being statistically significant (*p*s < 0.05). There was no significant change over time for the at-risk group in the mean number of expansion vocalisations (Figure 2). 

GLMM analysis showed an increase in the number of basic canonical utterances from 6 to 15 months of age averaged across both groups, *F*(3, 119) = 14.811, *p* < 0.001, η_p_ = 0.39. Overall, the TD group produced more canonical syllables than at-risk infants, *F*(1, 119) = 9.244, *p* = 0.003, η_p_ = 0.23. Pair-wise contrasts with adjusted p values showed significant group differences at 9 and 15 months, *t*(119) = 3.134, *p* = 0.002, *t*(119) = 2.346, *p* = 0.021, respectively, but not 6 and 12 months, *t*(119) = 0.962, *p* = 0.338, *t*(119) = 1.757, *p* = 0.081, respectively. The rate of increase in production of canonical utterances across time was greater for TD children compared to at-risk children, however, the interaction between group and time did not reach statistical significance, *F*(3, 119) = 2.498, *p* = 0.063, η_p_ = 0.06. Findings were similar for the number of basic canonical vocalisations. There was a main effect of time, *F*(3, 119) = 22.762, *p* < 0.001, η_p_ = 0.37, with number of basic canonical vocalisations increasing with age, especially from the 9- to 12-month time point. The group main effect was significant, *F*(1, 119) = 9.564, *p* = 0.002, η_p_ = 0.24, with contrasts showing TD children produced significantly more basic canonical vocalisations than at-risk children at 9 and 15 months, *t*(119) = 2.127, *p* = 0.035, *t*(119) = 2.684, *p* = 0.008, respectively, but not 6 and 12 months, *t*(119) = 1.045, *p* = 0.298, *t*(119) = 1.730, *p* = 0.086, respectively. However, the interaction between group and time, in this instance, was statistically significant, *F*(3, 119) = 3.189, *p* = 0.026, η_p_ = 0.07, confirming a greater increase over time in the number of basic canonical vocalisations for TD than at-risk children. 

Given none of the children produced advanced canonical forms at 6 months of age, this time point was excluded from analysis. Similar to the number of basic canonical utterances, there was an effect of time showing an increase in the number of advanced utterances from 9 to 15 months, *F*(2, 93) = 11.829, *p* < 0.001, η_p_ = 0.29. Although, numerically, the TD group produced more advanced utterances than the at-risk group, there was no group main effect, *F*(1, 93) = 3.292, *p* = 0.073, η_p_ = 0.10, and while the increase over time in advanced forms was greater for TD than at-risk children, the interaction between group and time was not significant, *F*(2, 93) = 1.976, *p* = 0.144, η_p_ = 0.05. For the number of advanced vocalisations, there was a main effect of time, *F*(2, 93) = 5.967, *p* = 0.004, η_p_ = 0.19, showing the number of advanced vocalisations increased from 9 to 15 months, and while the group main effect was not significant, *F*(1, 93) = 3.767, *p* = 0.055, η_p_ = 0.11, there was a significant interaction between group and time, *F*(2, 93) = 3.766, *p* = 0.027, η_p_ = 0.12. Contrasts showed no difference between groups at 9 months of age, *t*(93) = 0.309, *p* = 0.758. The TD group showed a significantly higher number of advanced vocalisations than the at-risk group at both 12 and 15 months of age, *t*(93) = 2.102, *p* = 0.038, *t*(93) = 2.606, *p* = 0.011, respectively, consistent with a greater increase in the production of advanced forms for TD children compared to children at risk of CP across the 9- to 15-month age range.

#### 3.1.3. Percentage of Protophones

**Figure 3:**
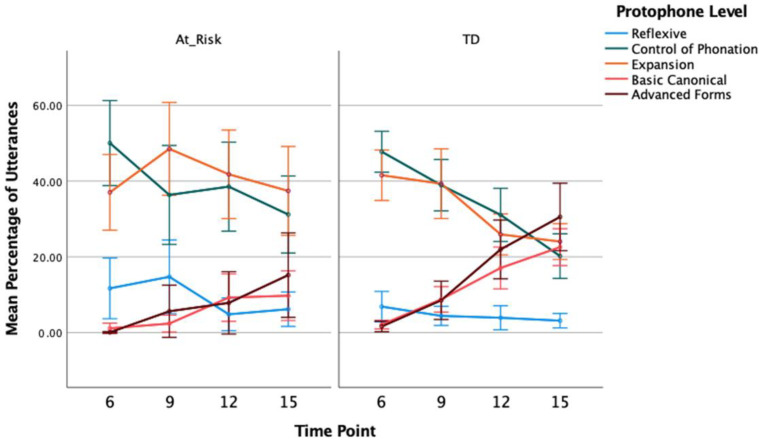
Mean Percentage (With 95% CI) of Total Infant Utterances Coded as Reflexive, Phonation Control, Expansion, Basic and Advanced Canonical for At-Risk and Typically Developing Children at 6, 9, 12, and 15 Months of Age.

Figure 3 (see, also, Table 3) shows the percentage of utterances coded at each protophone level according to the SAEVD-R for at-risk and TD groups at 6, 9, 12, and 15 months of age. 

GLMM analysis showed the percentage of reflexive utterances to be greater overall for at-risk compared to TD children, *F*(1, 119) = 4.296, *p* = 0.040, η_p_ = 0.12. Pair-wise contrasts showed the group difference was significant at 9 months, in particular, *t*(119) = 2.275, *p* = 0.025. The percentage of reflexive utterances reduced significantly across time, collapsed across both groups, *F*(3, 119) = 4.653, *p* = 0.004, η_p_ = 0.11, with the largest decrease occurring between 9 and 12 months. The interaction between group and time was not statistically significant, *F*(3, 119) = 2.163, *p* = 0.096, η_p_ = 0.07. These results were mirrored in the analysis of percentage of reflexive vocalisations, with the GLMM showing a main effect of group, *F*(1, 119) = 5.356, *p* = 0.022, η_p_ = 0.13, and time, *F*(3, 119) = 3.774, *p* = 0.013, η_p_ = 0.13, but no significant interaction between group and time, *F*(3, 119) = 1.782, *p* = 0.154, η_p_ = 0.05. Contrasts showed the group difference was only significant at 9 months with at-risk children showing a greater percentage of reflexive vocalisations, *t*(119) = 2.487, *p* = 0.014.

**Figure 4:**
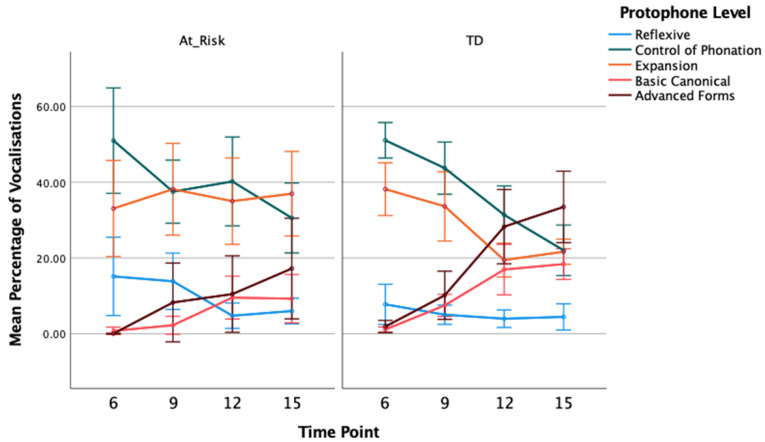
Mean Percentage (With 95% CI) of Total Infant Vocalisations Coded as Reflexive, Phonation Control, Expansion, Basic and Advanced Canonical for At-Risk and Typically Developing Children at 6, 9, 12, and 15 Months of Age.

There was a significant decrease in the percentage of control of phonation utterances across time (Figure 3), *F*(3, 119) = 9.989, *p* < 0.001, η_p_ = 0.30, but no difference between groups, *F*(1, 119) = 2.098, *p* = 0.150, η_p_ = 0.10, or interaction between group and time, *F* < 1. Again, these results were mirrored in the percentage of control of phonation vocalisations (Figure 4): there was a main effect of time, *F*(3, 119) = 12.886, *p* < 0.001, η_p_ = 0.37, but no group effect, *F* < 1, or group by time interaction, *F*(3, 119) = 1.863, *p* = 0.140, η_p_ = 0.06. 

For the percentage of expansion type utterances, there was a significant decline across time, *F*(3, 119) = 3.719, *p* = 0.013, η_p_ = 0.14, and the main effect of group showed the percentage of utterances coded as expansion was higher for the at-risk group than the TD group, *F*(1, 119) = 5.700, *p* = 0.019, η_p_ = 0.16. Contrasts showed group differences were significant at 12 and 15 months of age, *t*(119) = 2.724, *p* = 0.007, *t*(119) = 2.342, *p* = 0.021. Although the group difference was significant at later time points, the interaction between group and time was not significant, *F*(3, 119) = 2.333, *p* = 0.078, η_p_ = 0.09. For the percentage of expansion type vocalisations, there was a significant group effect with a higher percentage of expansions for at-risk compared to TD, *F*(1, 119) = 4.066, *p* < 0.046, η_p_ = 0.12. Contrasts also showed this group difference at 12 and 15 months only, *t*(119) = 2.816, *p* = 0.006, *t*(119) = 2.917, *p* = 0.004. There was no significant main effect of time, *F*(3, 119) = 2.258, *p* = 0.085, η_p_ = 0.09, and no interaction between group and time, *F*(3, 119) = 1.659, *p* = 0.180, η_p_ = 0.11. 

The percentage of basic canonical utterances increased across time, *F*(3, 119) = 20.899, *p* < 0.001, η_p_ = 0.45, and the TD group showed a higher percentage, overall, compared to the at-risk group, *F*(1, 119) = 16.045, *p* < 0.001, η_p_ = 0.33. The interaction between group and time was also significant, *F*(3, 119) = 4.212, *p* = 0.007, η_p_ = 0.10. The increase over time in the percentage of canonical utterances was greater for TD than at-risk children (Figure 3). While at 6 months the two groups did not differ, *t*(119) = 1.473, *p* = 0.143, the TD children showed a significantly higher percentage of basic canonical utterances at each subsequent time point, *t*(119) = 3.448, *p* = 0.001, *t*(119) = 2.060, *p* = 0.042, *t*(119) = 3.455, *p* = 0.001, for 9, 12, and 15 months, respectively. These results were replicated in the percentage of basic canonical vocalisations with significant effects of group, *F*(1, 119) = 11.854, *p* < 0.001, η_p_ = 0.28, time, *F*(3, 119) = 26.165, *p* < 0.001, η_p_ = 0.41, and group by time interaction, *F*(3, 119) = 4.019, *p* = 0.009, η_p_ = 0.06 (Figure 4). 

Excluding the 6-month time point, GLMM analysis of the percentage of advanced form utterances showed an effect of time, *F*(2, 93) = 15.459, *p* < 0.001, η_p_ = 0.33, with an overall increase in advanced utterances at each time point. While the TD group percentage was higher than that of the at-risk group when tested as a main effect, *F*(1, 93) = 6.473, *p* = 0.013, η_p_ = 0.17, there was also a significant interaction between group and time, *F*(2, 93) = 4.430, *p* = 0.015, η_p_ = 0.08. From a similar percentage at 9 months, the TD group showed a steeper trajectory with increasing age than the at-risk group. Contrasts confirmed there was no group difference at 9 months, *t*(93) = 0.747, *p* = 0.457, but the TD group had a significantly higher percentage of advanced forms at both 12 and 15 months of age, *t*(93) = 2.743, *p* = 0.007, *t*(93) = 2.370, *p* = 0.020, respectively. For the percentage of advanced vocalisations, there was also a significant group effect, *F*(1, 93) = 4.900, *p* = 0.029, η_p_ = 0.14, time effect, *F*(2, 93) = 12.312, *p* < 0.001, η_p_ = 0.28, and group by time interaction, *F*(2, 93) = 4.407, *p* = 0.015, η_p_ = 0.10, with significant group contrasts at the 12- and 15-month time points, *t*(93) = 2.779, *p* = 0.007, *t*(93) = 2.199, *p* = 0.030. As with the percentage of utterances, at-risk children produced a smaller percentage of advanced vocalisations at those two later time points. 

#### 3.1.4. Level of Infant Vocal Development

The criterion for determining the stage of vocal development of a child adopted by Nathani et al. [8] was applied to the present data. The highest protophone level where the child has an utterance percentage greater than 10% was taken to be that child’s current stage of development, and this was determined for each child at each time point (see Table 4). 

As can be seen in Table 4, most TD and all at-risk children were at the pre-canonical expansion stage of babbling at 6 months. At 9 months, most at-risk children remained at the expansion level, with more TD children (51%) having progressed to the canonical babbling stage (either basic or advanced forms level). Chi square analysis showed the number of children at the pre-canonical level (combining control of phonation and expansion) and canonical level (combining basic canonical and advanced forms) did not differ significantly between the at-risk and TD groups at 9 months, however, χ^2^(1) = 3.182, *p* = 0.074. 

By 12 and 15 months, most TD children had progressed to the advanced forms level while 67% of the at-risk infants at 12 months and 53% at 15 months remained at the pre-canonical stage. This group difference was statistically significant at 12 months, χ^2^(1) = 10.913, *p* < 0.001, and at 15 months, *p* = 0.003 (Fisher’s exact test). Six out of the fifteen at-risk children met SAEVD-R criteria for the advanced forms level of protophone development at 15 months of age, showing heterogeneity in the early speech production skills among the at-risk infants.

### 3.2. Is There Good Agreement between the IMP and the SAEVD-R in Identifying Departure from Typical Vocal Development in Children at Risk of Communication Impairment Secondary to CP?

#### Associations between the SAEVD-R and the IMP 

Spearman correlations for at-risk infants between the SAEVD-R measures at each time point and achievement in vocal development at 12 months of age using the IMP ceiling total score are in Table 5. Non-parametric correlations are reported because of violations to normality. There was no significant correlation between protophone type number or percentage at 6 months of age and the IMP ceiling score. Because of missing data, however, the sample size was relatively small (*n* = 10), thereby affecting statistical power. 

At 9 months, the number and percentage of basic canonical vocalisations and advanced forms showed a moderately strong positive correlation with 12-month IMP ceiling scores, indicating the degree to which an infant at risk of CP has progressed to the canonical stage of vocal development at 9 months of age is predictive of their outcomes on the IMP at 12 months of age. At 12 months, there was a strong positive correlation between the number and percentage of basic canonical and advanced forms with IMP ceiling scores, suggesting the amount of basic and advanced canonical vocalisations at 12 months can be highly predictive of scores on the IMP at 12 months of age. No correlation between the SAEVD-R measures at any time point and the 12-month IMP ceiling score was significant for the TD children. 

We used receiver operating characteristic (ROC) analysis to test whether the IMP was accurate in identifying delayed vocal development in at-risk and TD children at 12 and 15 months of age as assessed by the SAEVD-R. In particular, children at 12 and 15 months of age who remained at the pre-canonical stage (either control of phonation or expansion), based on their highest protophone level exceeding the 10% of utterances criterion, were classed as positive for vocal delay. Children who had progressed to the basic or advanced canonical stage at 12 and 15 months were classed as having age-typical protophone production (i.e., negative for delay). As shown in Table 4, 10 at-risk and 2 TD children had vocal delay at 12 months, and 8 at-risk and 1 TD child had vocal delay at 15 months. The area under the curve (AUC) assesses the overall accuracy of the test variable (12-month IMP total ceiling score), considering sensitivity and specificity, in classifying the children with and without vocal delay. Table 6 shows high levels of classification accuracy at both 12 and 15 months of age using the 12-month IMP scores for all children combined, and for the at-risk group separately, suggesting very good sensitivity and specificity. Accuracy in classifying vocal delay just for the TD children was poor, but this is unsurprising given only one to two TD children were positive for vocal delay at those time points and most TD children were at or close to the maximum IMP ceiling score at 12 months of age. The optimum cutoff point in the ROC curve when classifying all children was an IMP ceiling score of 13.5 at 12 months (sensitivity = 0.833, specificity = 0.905) and at 15 months (sensitivity = 0.889, specificity = 0.833). 

## 4. Discussion

The purpose of this study was to extend the findings of Ward et al. [21] to evaluate the validity of the IMP in identifying potential pre-linguistic markers of communication impairment through the human coding of infant vocalisations, captured through naturalistic sampling, via the Language Environment Analysis (LENA) digital language processor (DLP). The SAEVD-R [8] was used to characterise and analyse over 10,000 infant vocalisations in children identified as “at risk” of CP with age-matched typically developing (TD) infants to identify possible markers of early communication impairment across four time points (6, 9, 12, and 15 months of age). Two main aims were addressed: the first was to evaluate age-related changes in infant vocalisations by comparing differences in the number, percentage, and level of protophones, as measured using the SAEVD-R, in at-risk infants as compared with TD infants. The second aim was to further validate the clincal utility of the IMP in predicting early communication impairment by examining the relationship between level of vocal development using the SAEVD-R and 12-month outcomes using the IMP.

Our results indicate the TD and at-risk groups both demonstrated age-related changes in the number, percentage, and level of early vocalisations. Specifically, non-speech vocalisations (e.g., cries and vegetative sounds) progressively decreased with age, whilst speech-like vocalisations increased in volume and complexity. These findings are consistent with the literature reporting a universal pattern of vocal development [23,30]. In addition, our study identified group differences between at-risk and TD infants in the timing and progression towards more advanced vocalisations at 9 months through 15 months of age and, for at-risk infants, a strong association between the vocalisations coded using the SAEVD-R and 12-month outcome scores on the IMP. The study results for each research question will be discussed in turn.

### 4.1. Q1: Number, Percentage, and Level of Vocalisations 

At 6 months, the most frequently sampled vocalisations for both the TD and at-risk children alike were the non-speech reflexive and earlier-developing protophones coded at levels 2 (control of phonation) and 3 (expansion), including evidence of consonant–vowel sequences that were identified as marginal babble [8,9]. This is not unexpected, given our previous reported findings [21]. It is also concordant with the extant literature that considers early vocal development to be “grounded in our biological heritage” [41] (p. 1426). Per contra, significant group differences were observed in the overall number of infant vocalisations, suggestive of reduced volubility in the at-risk children.

Volubility, defined as the “number of speech-like vocalisations within a specified time period” [42] (p. 471), like canonical babble, is regarded as a robust phenomenon of infant development. Moreover, the literature has identified low volubility as a possible indicator of later language scores [42,43], with suggestions of a possible marker of communication impairment in some populations. For example, low volubility has been identified in children with fragile X [44] and childhood apraxia of speech [45] but not Down syndrome [16]. It is therefore possible that low volubility at 6 months and lack of progression in vocal development at 9 months could be useful clinical indicators of communication impairment.

By 9 months of age, group differences were evident in the complexity of infant vocalisations. Overall, the TD children showed a progression to more advanced forms of protophones with an increase in the number and percentage of level 4 basic canonical syllables and level 5 advanced forms observed across each time point. Collectively, these data indicate the TD infants in this study were building on previously established skills, replacing more primitive vocalisations with more advanced syllable forms, as is consistent with the age-based trends reported in the literature [8,10,46]. 

The children at risk of communication impairment secondary to CP, however, showed a slower trajectory of development. Their data show they remained at the expansion phase longer than the TD children, which suggests the ongoing use of expansion type syllables including marginal babble with fewer canonical forms. Further, their use of canonical babble and advanced forms appears to plateau between 12 and 15 months of age. These findings support our previous observation [21] and contribute to building the body of evidence showing children with CP differ in their ability to babble fluently and rhythmically [18,19,47]. 

Canonical syllables mark the transition to mature speech, with a critical window of emergence *before* 10 months of age [23,30]. They serve as the basic timing unit of language patterning [46] and result from the synchronous coordination of consonant and vowel gestures [48]. Nathani, Oller, and Neal [41] state “When infants begin to produce canonical syllables, they have achieved the motoric requirements to start learning to talk” (p. 1438). 

Kinematic studies of motor speech coordination indicate the progression to canonical babble and first words represents increasing motor control and mastery in the spatial and temporal coordination of vocal tract structures [49]. For example, Green et al. [49] examined the developmental trend in articulatory coupling and synchrony of the upper lip, lower lip, and jaw in a cross-sectional sample of infants, children, and adults, during the production of CV bilabial syllables. Age-related changes included TD infants (1-year olds) who presented with reduced spatial temporal coupling, characterised by excessive displacement and ballistic movements [49]. Increased coupling was evident at 2 years of age but characterised as rigid, differing from adult speakers who exhibited near-synchronous movements of the articulators that were characterised as highly specified and refined movements. These age-related biases and increases in spatial temporal coupling with age are consistent with increasing motor speech control.

A small body of evidence suggests children with motor speech impairment secondary to CP have poorer coordination of articulatory movements [50,51]. For example, in 2017, Nip [50] compared the spatial and temporal inter-articulator coordination between the jaw, upper lip, and lower lip across speaking tasks of 12 children (4–15 years of age) with spastic CP to 12 TD children. They identified decreased spatial coupling between the upper and lower lips and reduced temporal coupling between the jaw and lips for children with CP. They proposed the lack of inter-articulator coordination contributes to articulatory impairment.

In light of the above, it is proposed that the inability of the participants with CP in this study to transition from marginal to canonical babble and more advanced forms is suggestive of very early signs of timing and coordination difficulties, consistent with dysarthria [51,52]. Whilst further investigation of these observations is required, they do suggest the opportunity to identify the risk of motor speech impairment much earlier than current clinical practice. 

### 4.2. Q2: The Predictive Value of the IMP

Whilst the ecological validity of parent report measures is well recognised, human coding of vocalisation samples is considered the gold standard [53]. Our data, however, show strong correlations between vocal development at 9 and 12 months of age coded using the SAEVD-R and 12-month outcome scores on the IMP. Specifically, the amount of basic and advanced canonical syllables was found to be highly predictive of scores on the IMP at 12 months of age. ROC analysis also showed there was very good classification accuracy using the IMP 12-month outcome ceiling scores in identifying at-risk infants with delayed vocal development using SAEVD-R criteria at 12 months of age and also three years later at 15 months of age. Overall, our results suggest a lack or low frequency of canonical vocalisations (basic and advanced) combined with more pre-canonical vocalisations could be associated with poorer language outcomes in infants who are at risk of CP. Our findings, therefore, strengthen the clinical utility of the IMP as a predictive tool in detecting age-related changes in infant vocalisations and sensitivity to departure from typical development in children at risk of communication impairment secondary to CP.

Our findings have significant clinical implications. The ability to accurately identify early departure from typical development in infants at risk of communication impairment, without the need for labour- and resource-intensive human coding, provides clinicians with the means to assess and expediate access to targeted early intervention services much earlier than current practice. 

Clinical utility is nevertheless a multi-dimensional construct that addresses not only the meaningfulness and relevance of the information obtained but also fit with workplace practice in terms of ease of use, time, training and qualifications, format, and ease of interpretation [54]. The IMP is a validated norm-referenced, easy to administer, 16-item interview-based assessment of vocal development, with the first baseline assessment undertaken before 6 months of age. Self-instructional training in the administration and scoring is delivered online by the developer [28,36] and an electronic version is now offered (eIMP Online automatically calculates and reports stage-for-age trajectories and growth). The routine administration of the IMP as standard care within the at-risk clinic at Perth Children’s Hospital (paper in preparation) speaks to workplace utility of the IMP. The IMP, therefore, addresses many systems level challenges in the diagnostic process that include the requirement for specialist skills through multi-day training workshops (e.g., GMA). 

The absence of psychometrically robust assessment tools, formulated to predict early (i.e., ≤2 years) communication impairment secondary to cerebral palsy, including speech motor impairment, compels clinicians to rely on non-standardised and informal observations [55]. Whilst the sample size of this study is small, these early data suggest the IMP has a valid role in the tracking of early speech motor impairment in young infants and offers healthcare professionals the ability to benchmark an individual’s performance relative to peers and determine evidence-informed treatment objectives.

### 4.3. Limitations and Future Directions

The study has several limitations that warrant consideration. As reported by Ward et al. [21], the sample size is relatively small, with a bias of participants with spastic CP and dyskinetic CP. However, it is worth noting that the labour- and resource-intensive nature of hand coding often results in small sample sizes and our sample size is larger than has been reported in previous studies. For instance, Levin’s study [18] included 8 infants with CP, in Nyman et al.’s study [20] 6 out of 18 infants presented with CP, and Long and Hustad [19] reported on 7 out of 10 infants with/at risk of CP. Moreover, we have tracked infants longitudinally over four time points. Whilst small sample sizes limit generalisability, our data further contribute to an evidence base that posits that speech impairment in infants with CP may be evident in early infant vocalisations. 

All infant vocalisations were obtained by naturalistic sampling, within the home environment, with no consideration given to the communicative intent of the vocalisations. Further, it was not possible to observe occasions of mouthing and the impact on vocalisations. Despite this, the literature has acknowledged the richness of language sampling within the home environment, as contrasted with the research environment where recordings are typically short and lacking social context [22]. 

In our search for an early marker of speech motor impairment, we have utilised an assessment protocol grounded in motor control. However, as stated by Levin [18], it would be simplistic to view motor control as the only variable. It is acknowledged that delayed canonical babble may also be associated with cognitive–linguistic and environmental factors that are beyond the scope of those reported in this paper.

In view of these limitations, we are currently undertaking acoustic analysis of the audio data (infant vocalisations) to inform on timing and spectral characteristics of the canonical and pre-canonical protophones and further develop our understanding of more qualitative differences in articulatory control of infant vocal behaviours between infants at risk of CP and TD infants. Confirmation of communication impairment including diagnosis of speech motor impairment or dysarthria at an older age is also required to confirm the predictive value of different profiles of early vocal development in infants at risk of CP. 

## 5. Conclusions

The findings from this study extend our previous research [21] by showing through direct analysis of more than 10,000 infant vocalisation samples that children at risk of communication impairment secondary to CP did not progress to canonical babble within the first 12 months of age but produced predominantly pre-canonical expansion type sounds that would include production of vowels, vowel glides, and marginal babble. Further, a flatter trajectory of development was identified, with strong associations between performance on the IMP at 12 months and level of vocal development measured on the SAEVD-R.

Based on our fine-grained longitudinal analysis of the data, we conclude the IMP has validity and clinical utility in identifying early departure from typical development and risk of communication impairment. Further, the IMP interview format and scoring system provide clinicians with an easy to administer and cost-effective surveillance tool for earlier identification of the risk of communication impairment and referral to appropriate intervention services. 

Finally, our research makes a novel contribution by providing critically needed longitudinal analysis characterising the vocalisations of infants at risk of communication impairment, secondary to CP. Future research will focus on the development of growth profiles and trajectories characterising the development of vocalisations in infants with CP, based on motor profiles.

## Figures and Tables

**Table 1:** Participant Characteristics.

		At Risk	TD 18 (%)
		*n* (%)	*n* (%)
Sex	M	8 (53)	13 (72)
	F	7 (47)	5 (28)
CP motor type	Dyskinetic	7 (47)	-
	Spastic	5 (33)	-
	n/a	3 (20)	-
Distribution	Unilateral	1 (7)	-
	Bilateral	11 (73)	-
	n/a	3 (20)	-
MRI	*Timing of Insult*		
	Anti	3 (20)	-
	Post	1 (7)	-
	Peri	11 (73)	-
	*Description*		
	Grey	9 (60)	-
	White	5 (33)	-
	Maldevelopment	1 (7)	
Hearing impairment	WNL	13 (87)	15 (83)
	Aided (sensorineural loss)	2 (13)	-
	Episodes of otitis media	1 (7)	3 (17)
Oropharyngeal dysphagia		10 (67)	-
	*Feeding Method*		-
	Oral	11 (73)	18 (100)
	PEG	4 (27)	-

*Note*: WNL = within normal limits, PEG = percutaneous endoscopic gastrostomy tube, n/a = not applicable; M = male; F = female.

**Table 2:** Mean (Standard Deviation) Number of Utterances and Vocalisations in Total and at Each SAEVD-R Level for Infants at Risk of Cerebral Palsy (*n* = 15) and Typically Developing Infants (*n* = 18) At 6, 9, 12, and 15 Months of Age.

	Number Utterances				Number Vocalisations			
	6	9	12	15	6	9	12	15
Total								
At Risk	89 (22)	63 (19)	84 (30)	83 (30)	203 (57)	218 (115)	229 (95)	202 (73)
TD	85 (17)	76 (18)	81 (21)	89 (23)	279 (85)	221 (63)	254 (83)	293 (102)
Reflexive								
At Risk	11 (11)	8 (8)	4 (6)	6 (10)	30 (28)	30 (27)	13 (20)	12 (15)
TD	6 (7)	4 (4)	3 (6)	3 (3)	19 (25)	11 (12)	10 (12)	13 (20)
Control of Phonation								
At Risk	44 (15)	23 (15)	32 (21)	25 (21)	101 (40)	80 (47)	88 (56)	58 (41)
TD	41 (11)	31 (16)	24 (12)	18 (12)	143 (51)	100 (48)	75 (36)	60 (33)
Expansion								
At Risk	34 (16)	29 (12)	34 (23)	27 (15)	71 (50)	72 (50)	71 (44)	66 (33)
TD	36 (15)	30 (14)	21 (10)	21 (9)	109 (60)	72 (43)	50 (30)	63 (29)
Basic Canonical								
At Risk	1 (2)	2 (4)	8 (10)	10 (15)	2 (4)	6 (12)	23 (33)	24 (34)
TD	2 (2)	6 (4)	14 (11)	20 (10)	3 (4)	15 (11)	44 (38)	52 (28)
Advanced Forms								
At Risk	0 (0)	5 (11)	9 (18)	15 (23)	0 (0)	30 (75)	33 (63)	41 (62)
TD	1 (2)	6 (7)	18 (13)	28 (19)	5 (9)	23 (33)	76 (57)	106 (84)

*Note.* Sample size = 10 for at-risk infants at 6 months of age because of late entry into the research program.

**Table 3:** Mean (Standard Deviation) Percentage of Utterances and Vocalisations at Each SAEVD-R Protophone Level for Infants at Risk of Cerebral Palsy (*n* = 15) and Typically Developing Infants (*n* = 18) At 6, 9, 12, and 15 Months of Age.

	Percentage Utterances				Percentage Vocalisations			
	6	9	12	15	6	9	12	15
Reflexive								
At Risk	11.7 (11.2)	14.7 (17.6)	4.8 (7.8)	6.2 (8.2)	15.1 (14.4)	13.8 (13.4)	4.8 (6.1)	6.0 (6.1)
TD	6.9 (8.1)	4.4 (5.1)	3.9 (6.4)	3.2 (3.8)	7.8 (10.7)	5.0 (5.1)	4.0 (4.6)	4.4 (7.0)
Control of Phonation								
At Risk	50.0 (15.7)	36.4 (23.6)	38.5 (21.2)	31.2 (18.4)	51.0 (19.4)	37.5 (15.0)	40.2 (21.2)	30.6 (16.7)
TD	47.8 (10.9)	38.9 (13.6)	31.1 (14.1)	20.2 (11.8)	51.1 (9.4)	43.7 (13.8)	31.4 (15.4)	22.0 (13.4)
Expansion								
At Risk	37.0 (14.0)	48.5 (22.1)	41.8 (21.1)	37.4 (21.2)	33.1 (17.7)	38.2 (21.9)	35.0 (20.6)	37.0 (20.1)
TD	41.6 (13.4)	39.3 (18.5)	25.9 (10.9)	24.0 (9.5)	38.2 (14.0)	33.6 (18.4)	19.4 (9.0)	21.7 (6.7)
Basic Canonical								
At Risk	1.1 (1.9)	2.4 (4.0)	9.3 (11.3)	9.8 (11.8)	0.8 (1.4)	2.2 (4.3)	9.5 (10.2)	9.3 (11.5)
TD	2.1 (2.3)	8.8 (6.8)	17.1 (11.1)	22.6 (9.8)	1.1 (1.4)	7.5 (5.9)	16.9 (13.4)	18.4 (8.1)
Advanced Forms								
At Risk	0.1 (0.3)	5.6 (12.4)	7.9 (14.9)	15.2 (20.1)	0.0 (0.1)	8.3 (18.8)	10.5 (18.2)	17.2 (24.0)
TD	1.6 (2.8)	8.5 (10.2)	22.0 (15.6)	30.6 (17.9)	1.9 (3.3)	10.1 (12.8)	28.3 (19.7)	33.5 (19.0)

*Note*. Sample size = 10 for at-risk infants at 6 months of age because of late entry into the research program.

**Table 4:** Number of At-Risk and Typically Developing Infants at Each SAEVD-R Vocal Development Level at 6, 9, 12, and 15 Months of Age.

	6 Months		9 Months		12 Months		15 Months	
	At Risk	TD	At Risk	TD	At Risk	TD	At Risk	TD
Control Phon.	0	1 (6)	0	1 (5)	1 (7)	0	1 (7)	0
Expansion	10 (100)	17 (94)	12 (80)	8 (44)	9 (60)	2 (11)	7 (46)	1 (5)
Basic Canon.	0	0	0	2 (11)	2 (13)	3 (17)	1 (7)	1 (5)
Advanced	0	0	3 (20)	7 (40)	3 (20)	13 (72)	6 (40)	16 (90)

*Note*. Percentages are in brackets.

**Table 5:** Spearman Correlations Between Number and Percentage of Vocalisations at Each Level of the SAEVD-R (Ages 6, 9, 12 months) and IMP Total Ceiling Score at 12 Months for Infants at Risk of Cerebral Palsy.

	Utterances			Vocalisations		
	Age			Age		
**Measure**	**6**	**9**	**12**	**6**	**9**	**12**
Number						
Reflexive	−0.399	−0.138	−0.222	−0.531	−0.187	−0.112
Control of Phonation	0.409	0.340	−0.281	0.315	−0.271	−0.262
Expansion	0.019	−0.009	−0.445	0.049	0.031	−0.347
Basic Canonical	0.410	**0.527** *	**0.807** **	0.494	**0.568** *	**0.889** **
Advanced Forms	-	**0.635** *	**0.814** **	-	**0.649** *	**0.874** **
Percentage						
Reflexive	−0.519	−0.249	−0.102	−0.580	−0.502	−0.179
Control of Phonation	0.352	0.249	−0.300	0.290	−0.231	−0.513
Expansion	0.049	−0.037	−0.443	0.130	−0.169	−0.440
Basic Canonical	0.410	**0.591** *	**0.841** **	0.494	**0.579** *	**0.789** *
Advanced Forms	-	**0.635** *	**0.814** **	-	**0.649** *	**0.862** **

*Note*. Age refers to age when corresponding SAEVD-R measures were obtained. *n* = 15 (except at 6 months *n* = 10). * *p* < 0.05 two-tailed, ** *p* < 0.001 two-tailed.

**Table 6:** Classification Accuracy of Delayed Vocal Development at 12 and 15 Months of Age by the IMP Based on ROC Analysis for All Children and Separately for At-Risk and Typically Developing Children.

Group	AUC	SE	*p* Value	95% CI
12 Months				
All Children	0.873	0.079	<0.001	0.718 to 1.000
At Risk	0.940	0.060	<0.001	0.822 to 1.058
TD	0.313	0.159	0.240	<−0.001 to 0.625
15 Months				
All Children	0.907	0.078	<0.001	0.755 to 1.000
At Risk	0.946	0.059	<0.001	0.830 to 1.063
TD	0.324	0.214	0.409	−0.096 to 0.743

*Note*: ROC = receiving operating characteristic. AUC = area under the ROC curve. See text for SAEVD-R criteria used for delayed vocal development. IMP 12-month scores were used as the test variable for each ROC analysis. SE = Standard Error. TD = typically developing.

## Data Availability

Data are available on reasonable request and compliance with the Western Australian Child and Adolescent Health Service Human Research Ethics Committee requirements.
